# From data sharing to data publishing [version 2; peer review: 2 approved, 1 approved with reservations]

**DOI:** 10.12688/mniopenres.12772.2

**Published:** 2019-01-31

**Authors:** Jean-Baptiste Poline

**Affiliations:** 1 Montreal Neurological Institute and Hospital, McGill University, Montréal, QC, H3A 2B4, Canada; 2 Henry H. Wheeler, Jr. Brain Imaging Center, Helen Wills Neuroscience Institute, University of California, Berkley, CA, 94720, USA

**Keywords:** Data sharing, data publishing, FAIR principles

## Abstract

Data sharing, i.e. depositing data in research community accessiblerepositories, is not becoming as rapidly widespread across the life scienceresearch community as hoped or expected. I consider the sociological and cultural context of research and lay out why the community should instead move to data publishing with a focus on neuroscience data, and outline practical steps that can be taken to realize this goal.

Some research practices evolve rapidly. In the past few years, the number of preprints in BioRxiv has more than doubled every year, from 797 articles in 2014, 1601 in 2015, 4,295 in 2016, and already 10,819 posted in 2017. This is transformative, and is likely to redefine the publishing world in years to come - but an article on a preprint archive system is not considered as “published” until the content has been reviewed by community experts for correctness (and sometimes, unfortunately, for “importance”).

In this letter, data sharing, or data dissemination, is defined as making data available on the web such that these can be reused by others, without formal review and stamp of approval by the community. Data publishing is taken here as the release of data with peer review and an editorial process, and citable. The prototypical example of a journal publishing data is Scientific data. While any data can be shared, only some datasets will be deemed as sufficiently useful to be published.

Data sharing has recently become more widespread. In the following of the Taking as an example the field of brain imaging, initiatives such as the Human Connectome Project, the UK Biobank, INDI, ABIDE, OpenfMRI, and many others have made very large datasets available to the community (Poldrack & Gorgolewski, 2014; Poline *et al.*, 2012). The number of publications using these datasets is growing fast and poses some interesting questions on the re-analysis of the same datasets (Poldrack & Poline, 2015). The benefits of data sharing are numerous, but first and foremost accessible data increases the chance for reproducibility and replicability. The release of data is increasingly mandated by funding agencies, such as the Wellcome Trust (see for instance the 2015 report from the United Kingdom Academy of Medical Sciences, https://acmedsci.ac.uk/viewFile/56314e40aac61. pdf), but many researchers also individually recognize that they should be releasing data, since these are research products acquired under their stewardship for the progress of science or medicine, and not their “property”. Given the numerous compelling studies on the lack of statistical power in neuroscience and brain imaging (Button *et al.*, 2013; Poldrack *et al.*, 2017) and its possible role in the reproducibility crisis in life sciences, there is a very strong scientific incentive to make data accessible to the research community.

Nevertheless, data sharing does not seem to be taking over the world of biomedical or neuroscience research at a pace similar to the growth of preprint archiving systems. There are clear reasons for this. A key one is that data is often thought of as an asset in a competitive environment, which disincentives sharing. While an article is always written to communicate research results in a well established process of peer review, releasing data to the scientific community necessitates efforts beyond current practices for the data to be documented appropriately, and requires sustainable local or remote infrastructures capable of dealing with possibly large amounts of data. Data may also be sensitive, therefore needing additional ethical and legal aspects to be considered and implemented. Data sharing with all the necessary environment - in other words making data FAIR (Findable,Accessible, Interoperable, Reusable (Wilkinson *et al.*, 2016)) - is therefore often thought to be “too complicated” or “too costly”. Data are therefore most often not disseminated with all theinformation that would make these reusable.

While it is certainly true that this would require effort, it seems that the key issue is motivation (or lack thereof). To take an example, when a new research technique appears promising, laboratories will eagerly invest in material or human resources to adopt it. This may take months or even years and can necessitate large financial resources, new recruitments, and/or months of staff training. While extensive data sharing would likely radically change the efficiency and speed of science, this is not (yet) thought to be worth investing heavily in, except in a few laboratories or institutions, such as the Montreal Neurological Institute with its Open Science Initiative (Owens, 2016).

It is time that data publishing supersedes data sharing. Since researchers are happy to invest time and resources to publish their work, and gain recognition from their peers through these publications, publishing data articles -see De Schutter, 2010 for an early description- is a solution to increase the number of available well documented and citable datasets, for both fundamental and clinical research. A data article is a full description of a dataset for its future use in research, and should contain all necessary corresponding information making the data-set useful for a research community. Data articles are standard articles and therefore participate to the current publication infrastructure that tracks impact and increases visibility (indexing in bibliographical database) and is used – or misused - for research assessment (Gorgolewski *et al.*, 2013). Some research even show that data articles may have higher citation counts compared to conventional articles (Leitner *et al*., 2016).

In addition to solving - at least partly - for the motivation issue, data publishing elevates data to a first class research object because it is reviewed for its usability and usefulness by the research community. It brings the peer review process to data accessibility, technical documentation, provenance, ethical and legal aspects, and quality measurements, etc. Data acquisition and quality checks do require time, effort, years of expertise and are fundamental to any scientific result (other than simulation or theory), and therefore deserve the recognition associated with a publication. Data papers are citable, transforming the FAIR principles into FORCE (FAIR, Open, Research-Object based, Citable Ecosystem, Data Citation Synthesis Group, 2014).

## Some practical steps to further data publishing.

Not all datasets are “publishable”. Similarely to research findings, a dataset would need to reach a certain level of quality, which could be assessed in terms of data usefulness, quality, documentation, and curation. Whether a dataset is of sufficient quality is decided through the peer review process. But what do we need to do, as a community, to reconsider data acquisition, documentation and curation as critical activities, and therefore make these research objects publishable in peer reviewed venues?

- Researchers can today engage in *training* on the tools and standards required for efficient and adequate management and reuse of datasets (see for instance the ReproNim NIH-funded project and its online training module on FAIR data, http://www.reproducibleimaging.org/module-FAIR-data/), and these tools may vary depending on the specificities of the data themselves. Training could for instance target the use of a database system when these infrastructures exist, or the use of more lightweight solutions, such as DataLad, a project that adds a layer of meta-data on the git-annex distributed data versioning system. Training should at least cover the appropriate metadata for data description, the ethical and legal constraints linked to data accessibility and reuse, legitimate license and data usage agreements, and information on the rationales for data paper publishing.

- Universities and institutions themselves can step up their training proposal in this domain. While some online resources exist, formal courses are needed on the technical, legal and ethical, and sustainability aspects of data management, provenance documentation, citation, FAIR principles and their possibleimplementations in specific domains. All of these will eventually be part of the life scientist’s curriculum This dovetails with the evolution of a university’s school of information and libraries mission, as they become the new stewards of sustainable repositories and long term digital archiving – and likely, in the future, of scholarship e-communication. These new training components could therefore be established through partnerships with libraries and schools of information and proposed in most university schools and departments.

- Funding bodies have both a simple and critical role to play. They need to ensure that their funds are being used with maximum efficiency, and therefore *mandate* data release when possible. Already the Wellcome Trust and NIMH amongst others have taken steps in this direction for scientific, ethical, societal, and economical reasons.

- Publishers and editors can also implement practical steps, to establish “data articles” as a key article type. While an increasing number of journals are now requiring that data availability be the norm, not the exception (PLOS, F1000Research and Royal Society Journals, Scientific Data are are examples of journals with data sharing requirements eg http://journals.plos.org/plosone/s/data-availability, see also Allison *et al*., 2016), the data article has still to become mainstream. For instance, Scientific Data is dedicating its publication to data articles, but data articles will be mainstream when they will be available for authors in most journals. How to implement such a type of article will depend on the editorialdecisions. In general, it would be better to have data article writen before articles on analyses findings are published: this would help reviewers to check the data quality and appropriate use, as well as providing a useful resource for the community at large. What constitute a publishable data article is an editorial decision, but established and accredited community standards for documentation and dissemination when they exist should be enforced by journals to foster efficient reuse of data. Data paper would be most useful if they precede the publication of research findings and help scientists assess the quality of thedata before they are analyzed, but they will also be important for reuse of retrospective datasets.

- Last but not least, international organizations and scientific societies can establish and develop standards for repositories as well as for datasets (eg, how the metadata is represented). Already, some journals are vetting for some “acceptable” repositories based on the amount of available metadata and their long term sustainability, but we still often lack recognized criteria for what should be considered a well-documented and long term accessible dataset. The International Neuroinformatics Coordinating Facility (INCF) will certainly play a key role in establishing standards and best practices in neuroscience and is now becoming a certification body for neuroscience standards. Recently, INCF has started to endorsed standards such as BIDS (Gorgolewski *et al*., 2016), this will help the community to efficiently communicate and reuse brain imaging data. Standards in other domains of neuroscience, when adopted by a large community, should provide greater efficiency but also allow analyses impossible to perform in practice without standardization or harmonization such as mega-analyses.

Today there is an increase in the number of journals accepting neuroscience-focused data articles (e.g. Scientific Data, GigaS-cience, F1000Research, eNeuro, eLife, MNI Open Research, Wellcome Open Research), but they make only for a small proportion of the literature and of the acquired datasets. While data papers are still a novelty, they should be more and more recognized for what they are: first class research objects, firopo R,citable and re-usable building blocks of science. This transformative change of practice – and culture - needs to involve the entire research community: funding agencies, publishers, editors, and researchers. In the future, computationally readable metadata are likely to be used to automatically update, refine, in/validate or generalize results with machine findable datasets, profoundly changing the practice of science. Additionally, software and analyses scripts may also reach the stage of publishable research object category (Eglen *et al*., 2017), leading to a full-fledged reproducible and re-usable publication. Let’s not share data: let’s publish them.

## Amendments from Version 1

In this new version, I have clarified a number of points. First,I defined what I meant by data publishing in contrast to data sharing. Second, I have expanded on what would be required to move from data sharing to data publishing and on some of the publication aspects. Third, I have also clarified why neuroscience standards are needed for efficiency and for addressing questions that require integration of many datasets. I have also expanded the funding section to list the Canada First Research Excellence Fund.

See referee reports
